# Biomimetic Sniffing Improves the Detection Performance of a 3D Printed Nose of a Dog and a Commercial Trace Vapor Detector

**DOI:** 10.1038/srep36876

**Published:** 2016-12-01

**Authors:** Matthew E. Staymates, William A. MacCrehan, Jessica L. Staymates, Roderick R. Kunz, Thomas Mendum, Ta-Hsuan Ong, Geoffrey Geurtsen, Greg J. Gillen, Brent A. Craven

**Affiliations:** 1 Material Measurement Laboratory, National Institute of Standards and Technology, Gaithersburg, MD 20899, USA; 2 Chemical, Microsystem, and Nanoscale Technologies, Massachusetts Institute of Technology Lincoln Laboratory, Lexington, MA 02421, USA; 3 Division of Applied Mechanics, Office of Science and Engineering Laboratories, Center for Devices and Radiological Health, U.S. Food and Drug Administration, Silver Spring, MD 20993, USA.

## Abstract

Unlike current chemical trace detection technology, dogs actively sniff to acquire an odor sample. Flow visualization experiments with an anatomically-similar 3D printed dog’s nose revealed the external aerodynamics during canine sniffing, where ventral-laterally expired air jets entrain odorant-laden air toward the nose, thereby extending the “aerodynamic reach” for inspiration of otherwise inaccessible odors. Chemical sampling and detection experiments quantified two modes of operation with the artificial nose-active sniffing and continuous inspiration-and demonstrated an increase in odorant detection by a factor of up to 18 for active sniffing. A 16-fold improvement in detection was demonstrated with a commercially-available explosives detector by applying this bio-inspired design principle and making the device “sniff” like a dog. These lessons learned from the dog may benefit the next-generation of vapor samplers for explosives, narcotics, pathogens, or even cancer, and could inform future bio-inspired designs for optimized sampling of odor plumes.

The detection of trace contraband materials is rapidly evolving, with new sensor technologies such as those that utilize nanomaterials^1^,^2^,^3^,^4^, microfluidics^5^,^6^,^7^, advanced mass spectrometry^8^,^9^, Colormetric sensor arrays^10^,^11^,^12^,^13^, fluorescence quenching^14^,^15^,^16^, and microcantilevers^17^,^18^,^19^. While the development of next-generation sensors is of critical importance, the sampling component of these devices is considerably less developed and is an often-overlooked link in the chain of events that must occur for successful detection^20^. Traditional vapor-based sampling for explosives, for example, uses either a passive approach with stationary detectors that wait for the analyte to arrive (e.g. sensor arrays^21^), or a suction-based approach that continuously inspires air. The passive approach to vapor sampling relies on flow from the surrounding environment (e.g., buoyancy- or momentum-driven flow), a limitation where the sample must be presented to the detector. Suction-based techniques sample fluid by continuously drawing it toward the detectors from all accessible directions. While more versatile than the passive approach, suction-based techniques suffer from having a limited aerodynamic “reach,” characterized by an inability to sample fluid outside of the immediate vicinity of the detector inlet^22^.

The dog, one of nature’s best chemical detectors, does not suffer from the many of the limitations that plague current vapor detection technology. The remarkable olfactory ability of our canine companions is widely documented in the literature and continues to amaze (e.g., see refs 23, 24, 25, 26, 27). The use of dogs as trace chemical detectors is now widespread and routine in search and rescue^28^,^29^, explosives detection^30^, cadaver detection^31^, illicit drug detection^32^, and, more recently, in detecting diseases such as cancer^23^,^33^,^34^,^35^.

Unlike current vapor sampling technology, dogs actively sniff (e.g. inspire and expire) to acquire an odor sample. A canine sniff generally consists of an inspiration followed by an expiration that is cyclically repeated at a frequency of approximately 5 Hz^36^. The external aerodynamics of canine sniffing has been studied experimentally^22^,^37^ and with computational fluid dynamics^36^,^38^. In short, during inspiration air near the nose, within a spatial extent of approximately 1–2 cm, is drawn into each nostril with a laminar, hemispherical profile. During expiration, the vestibule and nostril act as a flow diverter, ejecting an expired air jet that is directed ventral-laterally (downward and to the side). When sniffing a surface, the warm expired air jets mechanically disturb and volatilize latent odorant, thereby liberating vapor that may then be inspired^22^,^36^. Additionally, the direction of the expired air jet from each nostril serves two primary functions when sniffing a surface: 1) it prevents the blow-off of odorant sample directly in front of the nostril (enabling it to be subsequently inspired), and 2) it entrains, or draws, air in front of the nose toward the nostril, effectively extending the aerodynamic reach of the nose over simple inspiration alone. This fluid dynamic mechanism for extending the aerodynamic reach of the dog’s nose was the source of bioinspiration that motivated this study.

Applying bio-inspired principles to chemical sensing is not new, but our use of bioinspired design based on the dog to enhance odor sampling efficiency is novel. Previous work using insects^39^,^40^,^41^ and arthropods^42^,^43^ focused primarily on plume sampling behavior during chemotaxis after the plume has already been located, and also on the role that an organism’s bilateral sensors play in chemotaxis^44^. Lessons learned in these studies have been used to aid in the development of automated plume tracking algorithms^45^,^46^,^47^. Previous work in mammals has focused on the behavior of dogs^36^,^48^,^49^,^50^, rodents^51^,^52^,^53^,^54^, and humans^55^ during scent tracking, odorant detection, or olfactory discrimination tasks. The external aerodynamics of scent acquisition have also been investigated in the same species – dogs^36^,^37^, rodents^56^, and humans^55^. Based on flow visualization studies of canine sniffing^37^, Settles^22^ first suggested the use of jet-assisted odorant sampling for artificial chemical sensing (e.g., using a method for augmenting aerodynamic reach that was first patented by Aaberg^57^). However, to our knowledge, no study to date has successfully incorporated bio-inspired active sniffing in a man-made device and quantified the influence on chemical detection performance.

The objectives of this study are four-fold: 1) to better understand the external aerodynamics of canine olfaction using an anatomically-similar 3D printed model of the dog’s nose; 2) quantify chemical detection performance for active sniffing versus continuous inspiration (akin to suction-based sampling used by current technology); 3) using the dog as a source of bioinspiration, design a custom bio-inspired inlet for a commercially-available handheld explosives vapor detector that facilitates jet-assisted active sniffing; and 4) compare the performance of the device for active sniffing versus standard suction-based sampling.

## Results

### Flow visualization of canine sniffing using a 3D printed dog’s nose

**Figure 1:**
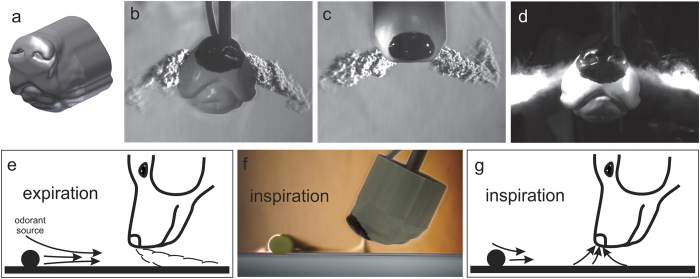
Jet-assisted fluid entrainment extends the aerodynamic reach of the canine nose during active sniffing. (**a**) Reconstructed model of the canine nose based on the model of Craven *et al*.^36^,^38^,^59^ (2007, 2009, 2010) that includes the nasal vestibule, external nose, lower jaw, and about 10 cm of the snout. (**b,c**) Images extracted from high-speed schlieren videography flow visualization with helium illustrate the directionality of the expelled air jets from anterior (**b**) and dorsal (**c**) views. (D) Visualization of theatrical fog shows the ventral-laterally directed turbulent air jets exiting the naris during expiration. (**e**) During the expiratory phase of sniffing, turbulent air jets vectored ventrally and laterally entrain odorant vapor from tens of centimeters ahead of the nose that would otherwise be inaccessible to the dog. (**f**) Schlieren image of the 3D printed dog’s nose during the inspiratory phase of sniffing showing acetone vapor be drawn into the nose from a source that is located approximately 10 cm away. (**g**) During the inspiratory phase of sniffing each nostril draws in air from all directions, including odorant-laden air that was drawn toward the nose during expiration. See Supplemental Information for video footage of these visualizations.

Using a combination of schlieren imaging and high-speed videography, we first visualized the external flow patterns associated with active sniffing of the 3D printed dog’s nose model (Fig. 1). The experiments revealed that, when the nose expires, a turbulent air jet exits each nostril and is vectored downwards (ventrally) and outwards (laterally) (Fig. 1b–d), as observed in previous experiments with live dogs^37^. The turbulent air jets act to entrain vapor-laden air from ahead of the dog’s nose and pull it toward the nostrils (Fig. 1g). This jet-assisted fluid entrainment increases the aerodynamic reach of the dog’s nose, drawing vapor-laden air toward the nostrils that would otherwise be inaccessible.

During the inspiratory phase of sniffing, air is uniformly drawn toward each nostril (Fig. 1e,f) with a limited aerodynamic reach that is characteristic of potential flow inlets^58^. This process of entraining vapor-laden air from extended distances during expiration and then quickly inspiring it is repeated at a rate of 5 times per second. These external flow patterns during active sniffing agree with previous experiments performed with live dogs^37^, which confirms our use of the present 3D printed dog’s nose model and a custom piston-cylinder assembly to mimic the external fluid dynamics of canine sniffing.

### Aerodynamic sampling performance of the 3D printed dog’s nose for active sniffing versus steady inspiration

**Figure 2:**
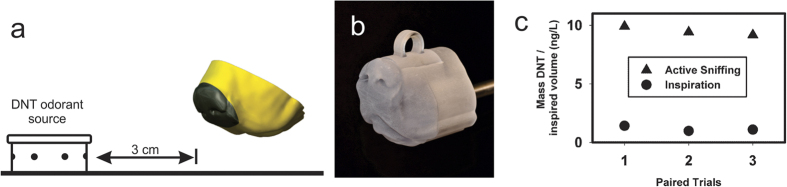
Aerodynamic sampling efficiency of the 3D printed dog’s nose during active sniffing versus steady inspiration measured via polyurethane foam (PUF) vapor collection with subsequent chemical extraction. (**a**) Schematic diagram illustrating the vapor source distance from the 3D printed dog’s nose model. (**b**) Photograph of the 3D printed nose that includes a removable PUF insert within the flow path in the vestibule of the nose that collects inspired DNT vapor. (**c**) Results from three paired trials that demonstrate an almost ten-fold increase in collected DNT vapor for active sniffing compared to steady inspiration. The average was 9.5 ± 0.37 ng/liter for active sniffing and 1.16 ± 0.23 ng/liter for pure inspiration.

Two different experimental approaches were used to directly compare the aerodynamic sampling performance of the 3D printed dog’s nose for active sniffing versus steady inspiration. The first approach utilized chemical extraction from a polyurethane foam (PUF) absorbent material placed in the vestibule of the dog’s nose model and subsequent analysis using liquid chromatography with ultraviolet detection to quantify the mass of 2,4-dinitrotoluene (DNT) collected from a uniform-release DNT vapor source^60^ (see Methods). The 3D printed dog’s nose model was placed 3 cm from the vapor source and 13 mm from the ground (Fig. 2a) and mechanically ventilated to mimic either active sniffing or steady inspiration with a peak flow rate of almost 60 L/min (see Methods). The aerodynamic sampling efficiency of DNT vapor, the primary signature odorant in trinitrotoluene (TNT) detected by canines^30^, was calculated as the total mass of DNT collected per unit volume of air inspired.

The results of the DNT sampling experiments reveal that the aerodynamic sampling efficiency of the 3D printed dog’s nose is approximately 8 times greater for active sniffing than for steady inspiration at a standoff distance of 3 cm from the DNT vapor source (Fig. 2c). That is, compared with steady inspiration, actively sniffing draws in roughly 8 times more DNT vapor per liter of air inspired. As shown in the flow visualization experiments (Fig. 1), this is due to the external aerodynamics of sniffing, where ventral-laterally directed air jets during expiration entrain and direct vapor-laden air toward the nose, which effectively extends the aerodynamic reach of the dog’s nose.

The second set of experiments to characterize the influence of active sniffing were performed by coupling the 3D printed dog’s nose directly to an ambient ionization mass spectrometer, enabling real-time monitoring of odorant signatures. The sniffing control system and inlet to the mass spectrometer shared the same flow path, which prevented exactly matching a physiological sniffing flow rate waveform (sinusoidal with a peak flow rate of approximately 48 L/min for the present 3D printed dog’s nose model that was reconstructed from high-resolution magnetic resonance imaging scans of a 29.5 kg mixed-breed Labrador retriever;^36^,^59^). Nonetheless, a representative sniffing flow rate consisting of a roughly sinusoidal waveform having a peak inspiratory flow rate of 25 L/min and peak expiratory flow rate of 53 L/min was used, while the mass spectrometer continuously subsampled 5 L/min from the time-varying airflow stream during both inspiration and expiration (see Methods).

**Figure 3:**
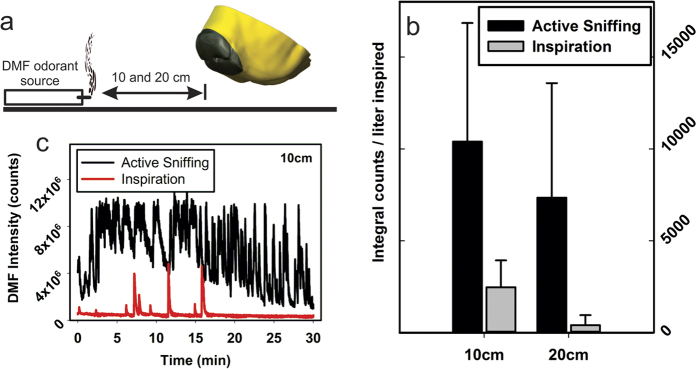
Sampling efficiency of DMF vapor by the 3D printed dog’s nose during active sniffing versus steady inspiration measured via ambient ionization mass spectrometry. (**a**) Illustration of the experiment. (**c**) Representative signal responses from the mass spectrometer comparing DMF signal intensity at 10 cm for active sniffing versus steady inspiration. (**b**) Total mass spectrometer response intensity, calculated as the integrated area under each curve, normalized by the total volume of inspired air for each experiment shows that active sniffing results in a significantly higher normalized response intensity compared with steady inspiration. The error bars are the standard deviation three sampling experiments for each case and are representative of the natural variance of the plume dynamics generated at the DMF vapor source. The improvements in DMF vapor detection performance (sniffing vs. steady inspiration) were approximately a factor of 4 at a standoff distance of 10 cm, and a factor of 18 at a 20 cm standoff distance.

A dimethylformamide (DMF) diffusion cell was used as the vapor source, with a calculated mass release rate of DMF vapor of 376 ng/min through a pinhole capillary. The vapor source was placed on axis with the dog’s nose model at two standoff distances (Fig. 3) and the model was mechanically ventilated to mimic active sniffing or constant inspiration, all while monitoring the DMF signal in the mass spectrometer (see Methods for details). The aerodynamic sampling efficiency of DMF vapor at both standoff distances from the nose was calculated as the integral under the DMF chromatograph curve per unit volume of inspired air (Fig. 3b).

The results of the DMF sampling experiments show that, under this specific set of experimental conditions, the DMF vapor sampling efficiency of the 3D printed dog’s nose is approximately 4 times greater for active sniffing than for steady inspiration at a standoff distance of 10 cm from the vapor source and roughly 18 times greater at a standoff distance of 20 cm (Fig. 3b). Again, this is due to entrainment from ventral-laterally vectored air jets during the expiratory phase of sniffing that act to draw odorant-laden air toward the nose that is subsequently inspired during the inspiratory phase of the sniff (Fig. 1, Figs S7 and S8).

### Bioinspired Sniffing Using a Commercial Trace Vapor Detector

**Figure 4:**
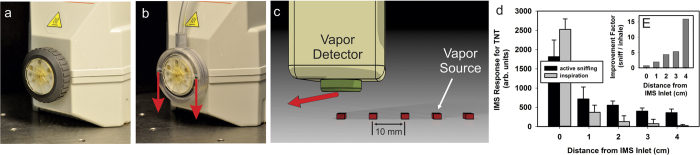
Performance of a commercial ion mobility spectrometer trace vapor detector (VaporTracer) outfitted with a custom bioinspired 3D printed inlet that enable the device to “sniff” in a manner similar to a dog. (**a**) Photograph showing the standard inlet of the VaporTracer. (**b**) Photograph of the custom 3D printed inlet and air supply line that replaces the conventional sampling inlet, enabling the VaporTracer to actively sniff. The red arrows illustrate the direction of the expired air jets that originate from holes on the sides of the inlet. (**c**) Schematic illustration of the experimental setup showing the detector inlet and the TNT vapor source^63^ (Staymates 2010), which was moved from directly beneath the inlet to 4 cm away from it, in 1 cm increments. The red arrow illustrates the direction of the expired air jets relative to the vapor source. (**d**) IMS response of the VaporTracer for TNT as a function of distance from the sampling inlet. Except when the vapor source is located directly beneath the detector inlet (where there is a marginal decrease in IMS response), active sniffing using the bioinspired inlet significantly enhances the IMS response for TNT at all sample locations compared with continuously drawing in air (the standard operating mode). Note that the volume of air that is analyzed by the IMS is the same for both sniffing and constant inspiration experiments. (**e**) Improvement factor calculated by dividing the IMS response for active sniffing by the associated response for continuous inspiration.

The previous experiments using the 3D printed dog’s nose demonstrate that active sniffing significantly extends the aerodynamic reach of the nose, permitting odorant acquisition over relatively large distances (up to 20 cm from the nose was demonstrated here). This suggests that a simple bioinspired modification of the inlet to current trace vapor detectors could be made to improve the sampling performance of the device. To test this bioinspired design concept, we retrofitted a commercially-available trace vapor detector (VaporTracer, Morpho Detection) based on Ion Mobility Spectrometric (IMS) detection^61^. This commercial device was fitted with a custom 3D printed inlet that was designed to mimic the external aerodynamics of the dog’s nose during active sniffing (Fig. 4a,b). Under normal operating conditions during sampling, the handheld IMS VaporTracer continuously draws in air for 10 seconds at a flow rate of approximately 1 L/min^62^, which, as previously discussed, limits the aerodynamic reach of the device. Using the custom bioinspired inlet, however, the VaporTracer is able to “sniff” like a dog, with a peak inspiratory flow rate of 10 L/min and a peak expiratory flow rate of 10 L/min at 5 Hz without modifying the normal operation of the IMS detection system. Importantly, expired air jets are vectored tangential to the inlet (Fig. 4b) to facilitate entrainment of vapor-laden air towards the inlet, as occurs during the expiratory phase of canine sniffing (see Fig. 1). The bioinspired inlet uses a separate mechanical sniffing system, so the total volume of analyzed air is the same for both active sniffing and inspiration-only experiments (see Methods for details).

To quantify the performance of the device using the bioinspired inlet and active sniffing compared with continuous air inspiration, experiments were performed with a continuous-release TNT vapor source^63^ positioned at 1 cm intervals from the inlet to the detector (Fig. 4c; see Methods). As shown in Fig. 4d, except when positioned directly beneath the inlet where the expired air jets disturb the vapor source, the IMS response for TNT is significantly greater (by a factor of up to 16) for active sniffing at all source locations. Active sniffing using the bioinspired inlet clearly extends the aerodynamic reach of the detector inlet, enabling odorant acquisition over a much larger distance compared with continuously drawing in air. As demonstrated here, with the exception of when the vapor source is located directly beneath the inlet, this simple modification significantly enhances the performance of the device.

## Discussion

Compared to continuous inspiration, active sniffing enhances the sampling performance of the dog’s nose by extending the aerodynamic reach of each nostril. Flow visualization experiments using a 3D printed dog’s nose revealed that during the expiratory phase of sniffing a ventral-laterally directed air jet exits each nostril that entrains vapor-laden air from in front of the nose and draws it towards the nostril (in some cases over distances exceeding 10’s of centimeters; see supplementary videos), where it is subsequently inspired. As demonstrated in the vapor sampling experiments, active sniffing enhances the sampling performance of the 3D printed dog’s nose by a factor of approximately 8 for 2,4-Dinitrotoluene (DNT) placed 3 cm from the nose, and by up to 18 times for dimethylformamide (DMF) located as far away as 20 cm from the nose. The discrepancies in performance improvement for these two materials may be partially attributed to their different densities (DMF being almost 2.5 times heavier than air and DNT being more than 6 times heavier than air), as the aerodynamic and fluid entrainment behavior of each will be slightly different.

Leveraging the external aerodynamics of canine sniffing as a source of bioinspiration, we fabricated a custom bioinspired inlet for a commercially-available vapor detection system (VaporTracer, Morpho Detection) that was designed to mimic the aerodynamic principles utilized by the dog to extend the aerodynamic reach of its nostril. That is, the custom 3D printed inlet permitted the instrument to effectively “sniff” like a dog without interfering with the normal operation of the device. Experiments demonstrated that, compared with continuously drawing in air (the normal operating mode of the device), active sniffing using the bioinspired inlet significantly extends the aerodynamic reach of the detector inlet, enabling acquisition and detection of TNT vapor at distances where the odorant is otherwise inaccessible.

As in the dog, the increased aerodynamic reach afforded by active sniffing using the custom bio-inspired inlet is not omnidirectional. That is, the aerodynamic reach is extended only in the direction opposite to the direction of the expired air jets. As demonstrated in the present study (see Fig. 4(d)), diminished detector performance was observed when the sample was located directly beneath the detector inlet due to disturbance of the vapor source by the expired air jets. Further reduction in performance is to be expected as the odor source is moved in the direction of the vectored airstream, until a complete loss of signal occurs when the air jets blow all vapor emanating from the source away from the detector inlet. In the dog, these flow patterns have several potential benefits. First, an increase in the aerodynamic reach in the anterior direction is beneficial for odor detection as the dog walks in the forward direction. This is an operational benefit that allows the detector (in this case the dog) to sense the presence of an odor from greater distances and further augmented with anterior directionality, thereby increasing the effective areal coverage rate of the detection system. Second, the warm expired air jets disturb an odor source and volatilize latent odorant that may then be inspired^22^,^36^. Taken together, the external flow patterns that develop during sniffing are likely one of the reasons that dogs have been observed to “scan” a ground plane in the anterior-posterior direction when sniffing a scent source^37^.

The principle of bioinspired sniffing based on the canine that was leveraged in this study could also be used in other chemical trace detection devices by retrofitting a similar bio-inspired inlet. Next-generation vapor trace detectors might also incorporate a bellows-style fluid pumping mechanism (e.g., see refs 22 and 64) to induce sniffing and possibly improve the sampling performance of the device. Future biomimetic devices that incorporate sniffing might also consider additional bio-inspired design principles based on the dog that include: 1) heating the expired air jets to volatilize latent odorant, and 2) operating the device using a “scanning” motion in the forward-aft direction that is aligned with the direction of the expired air jets (e.g., see ref. 37). Such modifications might further enhance the performance of the device. Importantly, as demonstrated in this study, leveraging such bio-inspired design principles for improved vapor sampling and detection performance of man-made devices does not require replicating the anatomy of the dog’s nose, but only the relevant geometrical features and the underlying physical mechanism of enhancement-in our case, the direction and pulsatile nature of expired turbulent air jets during sniffing.

Several limitations in the current study must be mentioned. First, the 3D printed dog’s nose included static nostrils, whereas in reality, a dog’s nares are mobile during sniffing^22^,^37^. Even so, our flow visualization experiments revealed the same external flow patterns as observed in live dogs^37^. This is further supported by the computational fluid dynamics (CFD) study of Craven *et al*.^36^, which included static nostrils and observed the same external aerodynamics during sniffing. Additionally, due to limitations of the experimental setup, in our experiments that directly coupled the 3D printed dog’s nose model to an ambient ionization mass spectrometer, we were unable to exactly match the physiological sniffing flow rate waveform. Nonetheless, a representative sniffing flow rate was used that further confirmed that the external aerodynamics of canine sniffing enhances vapor sampling efficiency compared with continuous inspiration. Moreover, the results demonstrated that the increase in aerodynamic sampling efficiency afforded by sniffing is likewise obtained using a peak inspiratory airflow rate that is lower than the nominal physiological value (see Results and ref. 36). Finally, the vapor sources used in the chemical detection measurements were very different, making it difficult to directly compare the PUF and mass spectrometry results because 1) the standoff distances were different, 2) the density of each chemical was different (DMF is about 2.5 times heavier than air and DNT is almost 6.3 times heavier than air), 3) the chemical analysis techniques were very different, 4) and the release rate of vapor from each source was different. It is possible that the DNT vapor source exhibited an increased release of vapor due to convection over the PDMS surface. However, we suspect that this did not significantly influence the results, as the low concentration of DNT within the PDMS matrix created a diffusion-limited case for mass transport of DNT to the air rather than one dominated by convective forces (see refs 60,65, 66, 67).

Future work may include incorporating additional bio-inspired design principles based on the dog into the retrofitted commercial trace vapor detector, namely including the use of heated air jets during expiration to volatilize latent odorant, and operating the device using a biomimetic scanning motion (see above). Additionally, future research may investigate stereolfaction^36^,^38^,^55^,^68^,^69^,^70^,^71^ of the canine by connecting a separate mass spectrometer to each nasal airway in the 3D printed dog’s nose model and using experiments and CFD to investigate the spatiotemporal nature of odorant acquisition in realistic odor environments to better understand how dogs track odor trails and localize an odorant source. Finally, future work may explore the role of air-jet assisted fluid entrainment on micro-particle removal and collection in the dog’s nose. While the current study focused on vapor sampling and detection, there continues to be unanswered questions regarding particle sampling versus vapor sampling with canines^22^,^37^.

In summary, the main points of this study are:

1. Several flow visualization techniques were used to investigate the external aerodynamics of canine olfaction during biomimetic sniffing with a 3D printed dog’s nose.
2. The vapor sampling performance of the 3D printed nose was evaluated with two chemical detection techniques to quantify the differences between active sniffing and constant inspiration.
3. Active sniffing (repetitive inspiration then expiration) increases the amount of analyte drawn towards the dog by almost a factor of 18 in some cases, compared to constant inspiration.
4. The development of a bio-inspired inlet on a commercially-available vapor detector created a system that “sniffed” like a dog.
5. Measurements with the bio-inspired inlet showed an improvement in analyte detection by a factor of up to 16 while the commercially-available vapor detection system was sniffing, compared to constant inspiration.
6. Bio-inspired design principles learned from the dog may be used to improve the performance of next-generation vapor detection technology.

## Methods

### Schlieren imaging

In the single-mirror schlieren imaging system, a diverging light beam passes through the test area where refractive index gradients bend the light away from their coincident path. The light then fills a spherical mirror and returns to a beam splitter that steers the beam as it approaches its focal point. A razor blade is positioned exactly at the focal point of the beam where a fraction of the bent light is cut-off, creating an image with light and dark contrasts representative of the density gradients in the test section. Here we use a single-mirror coincident beam schlieren system with a spherical mirror of 40.6 cm diameter with a focal length of 243.8 cm. A high-definition video camera captures video footage during the experiments. The vapor source (a foam plug) in Fig. 1 is about 10 cm away from the tip of the nose.

### 3D printing

The 3D printed dog’s nose was based on the model of Craven *et al*.^36^,^38^,^59^ that was reconstructed from high-resolution magnetic resonance imaging scans of a female mixed-breed Labrador retriever. The 3D printed model was fabricated on a Connex 500 digital materials 3D printer (Stratasys.com). No attempt was made to recreate the complex interior structures within the nose (e.g., see ref. 59), as this study is primarily interested in the external fluid dynamics during inspiration and expiration. Only the main external features of the nose need to be replicated to understand the most basic first-order attributes of its sampling behavior.

The additive manufacturing system uses an inkjet printing approach to create physical models from a polymeric resin material. The resin, initially in a liquid state, is deposited one cross-section at a time in 20 μm thick layers by a series of inkjet nozzles. The resin is a photopolymer that hardens upon photo initiation via ultraviolet light. Once a layer of liquid resin is deposited, a high-intensity ultraviolet light bombards the surface and cures the layer. Another layer is then deposited and the process is repeated. A gel-like support material is deposited in locations where future voids will exist. This support material is manually removed with a high-velocity water jet once the build is complete. A major benefit of this particular instrument is its ability to use two different resin materials, with different material properties (i.e. flexible vs. rigid), within the same printed part. This enables the creation of 3D parts with varying material properties, i.e. different colors or shore hardness values. Models printed with this technology possess very high resolution features that approach 40 μm in scale.

### Custom sniffing system

A custom piston/cylinder device^72^ was used to enable realistic sniffing for flow visualization experiments. This system was designed based on hot-wire anemometry measurements of Craven *et al*.^36^ of airflow during sniffing in several dog breeds. Specifically, their work provided the sniff frequency (5 Hz), airflow rate waveforms, and amplitudes that were utilized in this study.

A hot-wire anemometer was used to measure the flow rate of the piston/cylinder device, and ultimately the dog nose, as a function of time. The probe was positioned within the tubing that led to the dog nose, providing real-time measurement data *in-situ*. Since the hot-wire device has almost zero pressure drop, this measurement technique had little effect on the flow in the system resulting in accurate and time-resolved flow rate data. The hot-wire was calibrated with a mass flow controller (model FMA5542A, Omega.com) The response time of the probe was on the order of 1 ms, enabling transient changes in flow rate to be resolved as the dog nose model inhales and exhales.

For PUF measurements, the flow control system consisted of a vacuum pump, solenoid valve, and air pressure supply. The vacuum pump was continuously operating at approximately 60 L/min as the solenoid valve pulsed pressurized air at 5 Hz through the nose. The air pressure was regulated such that the flow rate exiting the nostrils of the nose was also approximately 60 L/min, creating a sniffing waveform that approaches that of a Labrador retriever. Shielding was used to minimize room currents during sampling experiments.

### Polyurethane foam measurements

The vapor source consisted of a shallow cylindrical metal tin can 78 mm × 28 mm with six 2 mm holes in the top and six 2 mm holes in the side with a 2 mm thick layer (10 g) of polydimethylsiloxane (PDMS, 93–500, Dow Corning, Midland, MI) thoroughly mixed with 1% mass fraction of 2,4-dinitrotoluene (DNT). This vapor-release device has been shown to provide a uniform vapor concentration of DNT for short-duration use^60^.

The 3D printed dog’s nose was modified to incorporate a polyurethane foam (PUF) adsorbant in the nasal vestibule (see Fig. 2b). A cylindrical cavity was created in the 3D dog nose that exactly accommodated a 22 mm × 36 mm PUF pad (precleaned ORBO 1000, cut in half, Sigma-Aldrich, St. Louis, MO, USA) that was further cleaned with acetonitrile. The dog’s nose model was placed 3 cm from the vapor can and 13 mm from the ground. A clean PUF insert was placed into the dog nose and then the mechanical sniffing system was activated, quantitatively collecting the DNT vapor on the adsorbant PUF surface. The PUF measurement duration for each trial was 20 minutes.

An internal standard (IS), 3,4-DNT was used to quantitate the 2.4-DNT by inserting the PUF into a glass air sampling tube (Sigma) with an added stopcock. The IS was introduced onto the incident surface of the PUF dissolved in 25 μL of a highly volatile solvent, 2-methylbutane (2-MB). The 2-MB was removed by flow of N_2_ though the PUF prior to elution. The accumulated DNT and IS were then eluted from the pad with two volumes (25 and 10 mL) of hot acetonitrile (50 °C) which were combined and concentrated under N_2_ prior to analysis. For determination of the 2,4-DNT, liquid chromatography with ultraviolet detection (LCUV) was used with a 2-pump gradient elution system with fixed wavelength detection at 230 nm. The chromatography column was a C-18 column (MAC MOD Analytical, PA ACE 3 C18, 150 × 3.0 mm, 3 μm particle with an acetonitrile:water linear gradient starting at 20% acetonitrile to 26% over 22 min and then held for an additional 8 min with a flow rate of 0.75 ml/min.

### Ambient Ionization Mass Spectrometry measurements

The diffusion cell consisted of a small glass vial (5 mm diameter, 20 mm length), a threaded nylon cap, and an o-ring. A hole was drilled through the nylon cap and a controlled leak was formed by gluing a 1.4 cm × 700 micrometer ID capillary tube into the hole using torr-seal.

The mass spectrometer used was an AB Sciex QTrap 5500 (sciex.com) coupled with a custom inlet source designed for secondary electrospray ionization (SESI)^73^,^74^. Operation was conducted in positive mode, and the m/z 74 to 46 DMF transition was monitored via multiple reaction monitoring. The SESI solvent was 70:30 methanol:water + 2% acetic acid.

The combination of mass spectrometer inlet flow rate (5 L/min) and a vacuum pump operating at 20 L/m provided an inspiratory flow rate of 25 L/min during constant inhalation experiments. During active sniffing, the flow followed a roughly sinusoidal waveform with a peak inspiratory flow rate of 25 L/min and peak expiratory flow rate of 53 L/min. In each case, the mass spectrometer was subsampling 5 L/min from the main airflow stream. See supplementary information Fig. S6 for a schematic diagram of the experimental setup.

The 3D printed dog’s nose model was placed 10 mm above a flat fluorocarbon polymer surface. Data were collected at 10 cm and 20 cm from the vapor source with 3 repeats at each location. Data acquisition time for each location was 30 minutes. A 3-minute background was acquired before each data collection event. After each experiment, the area was rinsed with isopropyl alcohol, fanned to clear residual vapors, and an additional 15 min was allowed to pass prior to the next experiment. Uncontrolled room currents played a surprising role in depleting the vapor signal, so all local air vents and return ducts were sealed during these experiments.

### VaporTracer ion mobility spectrometer

The inlet to the Vapor Tracer ion mobility spectrometer (IMS) (Morpho.com) was positioned 10 mm vertically from the flat surface. The vapor source consisted of a 10 mm diameter and 10 mm tall can with approximately 10 g of gelatin with 0.1% TNT concentration by weight. The TNT release rate was not measured, but previous work has shown that the rate is constant^63^. The IMS was functioning in a standard operating mode with a tube temperature of 180 °C, an inlet flow rate into the detector of 1 L/m, and a 10 s sampling duration. The mechanical ventilation system operated independently of the inlet flow to the IMS and did not change the overall amount of air sampled during the 10 s sampling period, for either active sniffing or constant inspiration.

Five replicate measurements were performed at each position of the vapor source and for each sampling mode (sniffing vs. inhale only). The vapor source was capped between each experiment to prevent undesired loss of TNT and to prevent vapor contamination on surrounding surfaces. The area was rinsed with ethyl alcohol between individual experiments.

### Disclaimer

Certain commercial equipment, instruments, and materials are identified in this work. Such identification does not imply recommendation or endorsement by the National Institute of Standards and Technology, Massachusetts Institute of Technology Lincoln Laboratory, the Food and Drug Administration, or the Federal Government, nor does it imply that the products identified are necessarily the best available for the purpose.

## Additional Information

**How to cite this article**: Staymates, M. E. *et al*. Biomimetic Sniffing Improves the Detection Performance of a 3D Printed Nose of a Dog and a Commercial Trace Vapor Detector. *Sci. Rep.* **6**, 36876; doi: 10.1038/srep36876 (2016).

**Publisher’s note**: Springer Nature remains neutral with regard to jurisdictional claims in published maps and institutional affiliations.

## Supplementary Material

Supplementary Figure S1

Supplementary Figure S2

Supplementary Figure S3

Supplementary Figure S4

Supplementary Figure S5

Supplementary Information
