# Supplementary material for: Biomimetic Sniffing Improves the Detection Performance of a 3D Printed Nose of a Dog and a Commercial Trace Vapor Detector
Source: Sci Rep. 2016 Dec 1;6:36876. doi: 10.1038/srep36876 (PMC5131614; doi:10.1038/srep36876)
Supplement: Supplementary Information [file srep36876-s6.pdf]

Biomimetic Sniffing Improves the Detection Performance of a 3D Printed Nose of a Dog and a Commercial Trace Vapor Detector

Matthew E. Staymates*[1], William A. MacCrehan[1], Jessica L. Staymates[1], Roderick R. Kunz[2], Thomas Mendum[2], Ta-Hsuan Ong[2], Geoffrey Geurtsen[2], Greg J. Gillen[1], and Brent A. Craven[3]

[1]Material Measurement Laboratory, National Institute of Standards and Technology, Gaithersburg, MD

[2]Chemical, Microsystem, and Nanoscale Technologies, Massachusetts Institute of Technology Lincoln Laboratory, Lexington, MA

[3]Division of Applied Mechanics, Office of Science and Engineering Laboratories, Center for Devices and Radiological Health, U.S. Food and Drug Administration, Silver Spring, MD

Supplementary Information:

Supplementary Information:

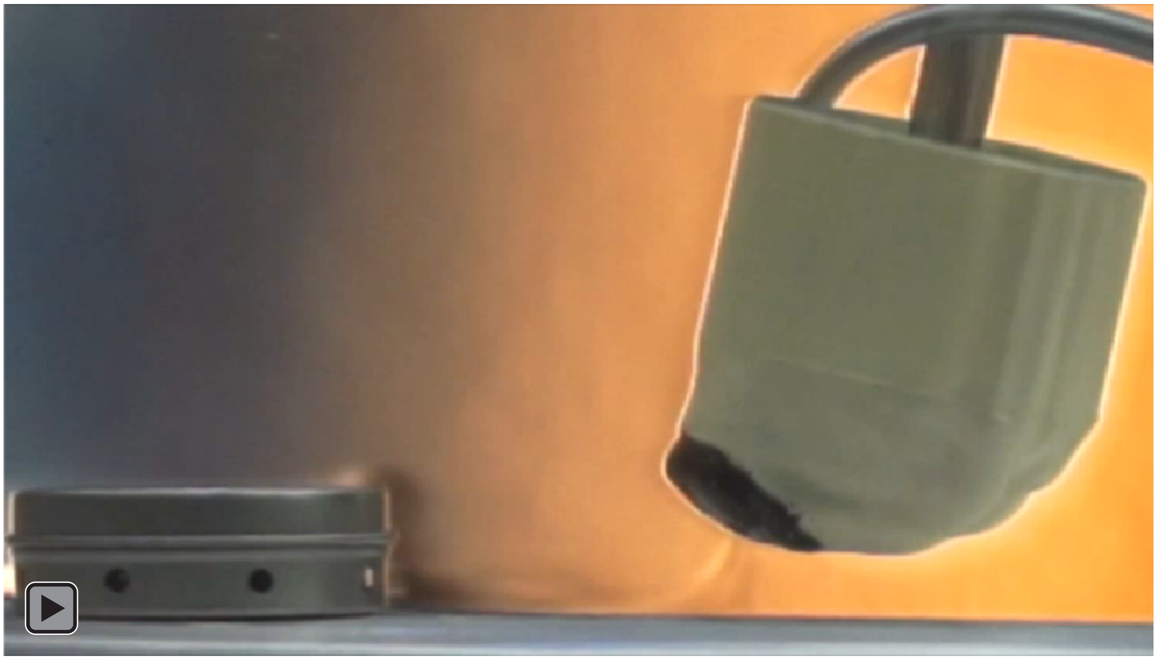

Figure S1. Schlieren footage of the biomimetic dog's nose sniffing an acetone-soaked foam plug within a training aid can. The frequency of sniffing is 5 Hz and the sinusoidal flow profile is similar to that of a female Labrador retriever. Click on the video to begin playing.

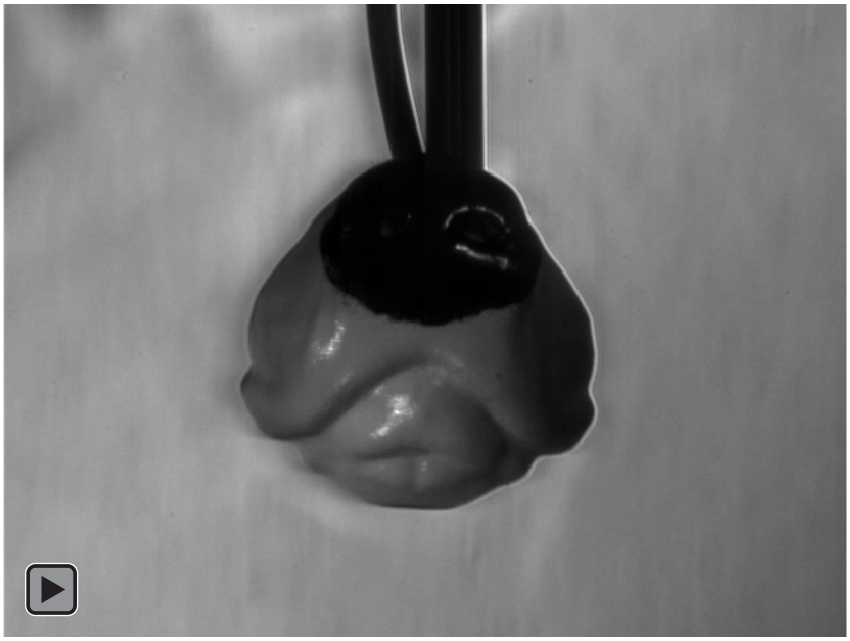

Figure S2. High-speed schlieren videography of the dog's nose exhaling helium in a schlieren optical system (anterior view). This video was filmed at 3000 frames per second and demonstrates the turbulent nature of the expired air jets that emanate from each nostril during exhale.

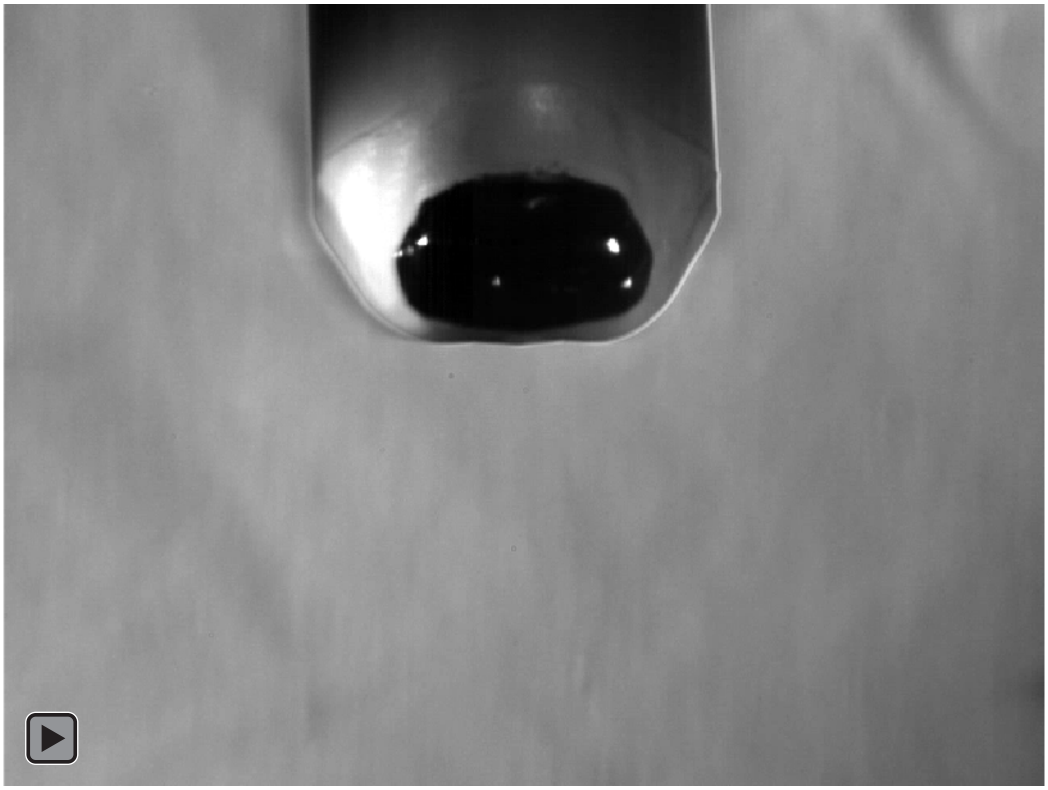

Figure S3. High-speed schlieren videography of the dog’s nose exhaling helium in a schlieren optical system (dorsal view). This video was filmed at 3000 frames per second.

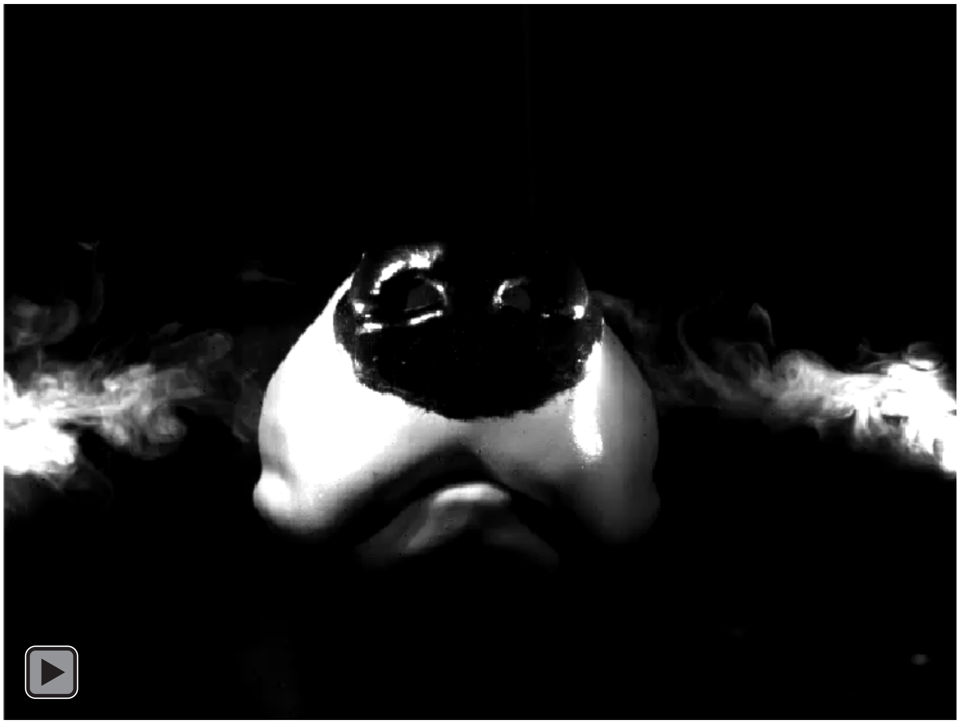

Figure S4. High-speed videography of the dog’s nose exhaling theatrical fog (anterior view) shows similar turbulent air jets originating from the nostrils during expiration. This video was filmed at 3000 frames per second.

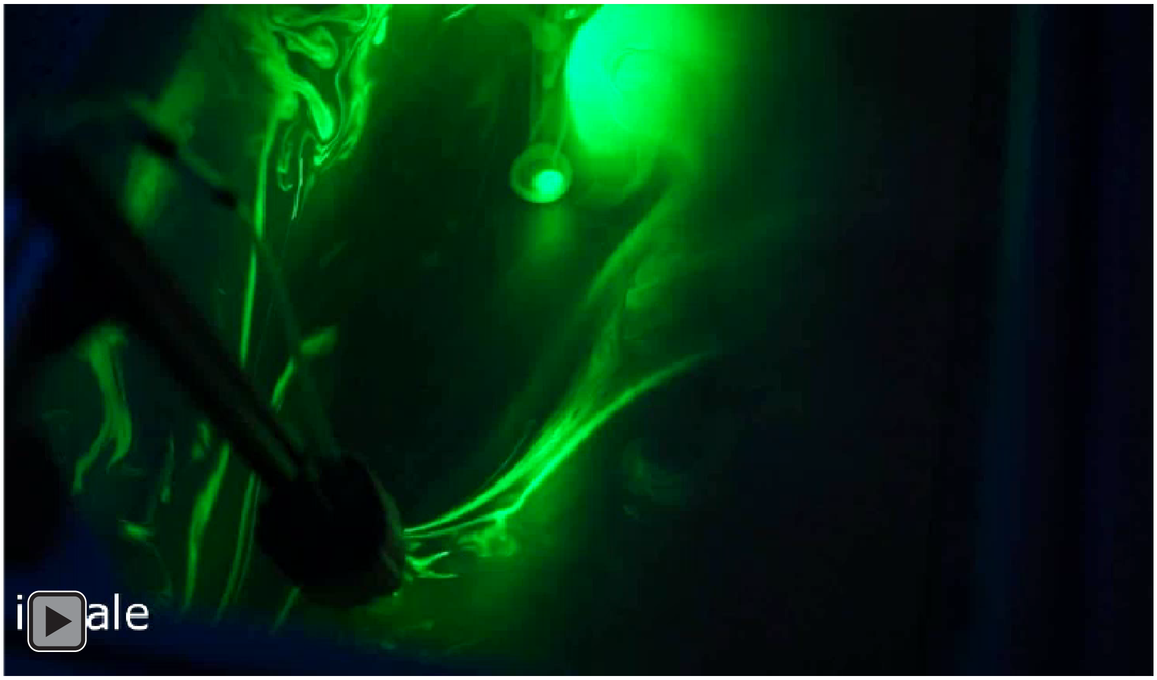

Figure S5. Laser light sheet flow visualization of the dog's nose sampling low-lying theatrical fog. The viewpoint in this video is looking over the right shoulder of the dog as it sniffs fog on a horizontal surface. The artificial dog's nose switches between active sniffing and constant inhalation to demonstrate the differences in the surrounding fluid dynamics as the dog sniffs. During active sniffing, flow is pulled from tens of centimeters in front of the nose, while during inhalation the aerodynamic reach of the nostrils is dramatically reduced.

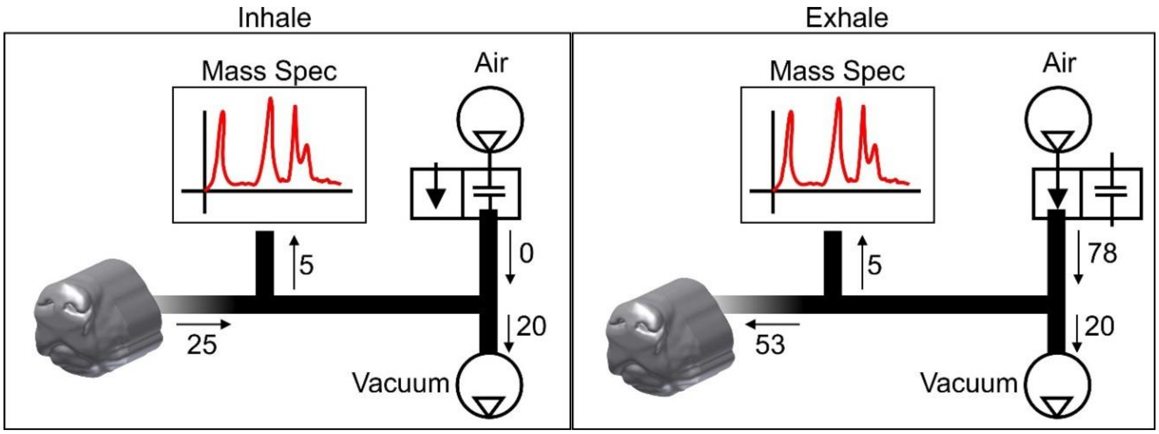

Figure S6. Schematic diagrams of the approximate flow rates during a sniffing cycle. The values shown here are estimates and were not measured directly, except at the air pressure source where a hot wire anemometer was used to measure flow rate.

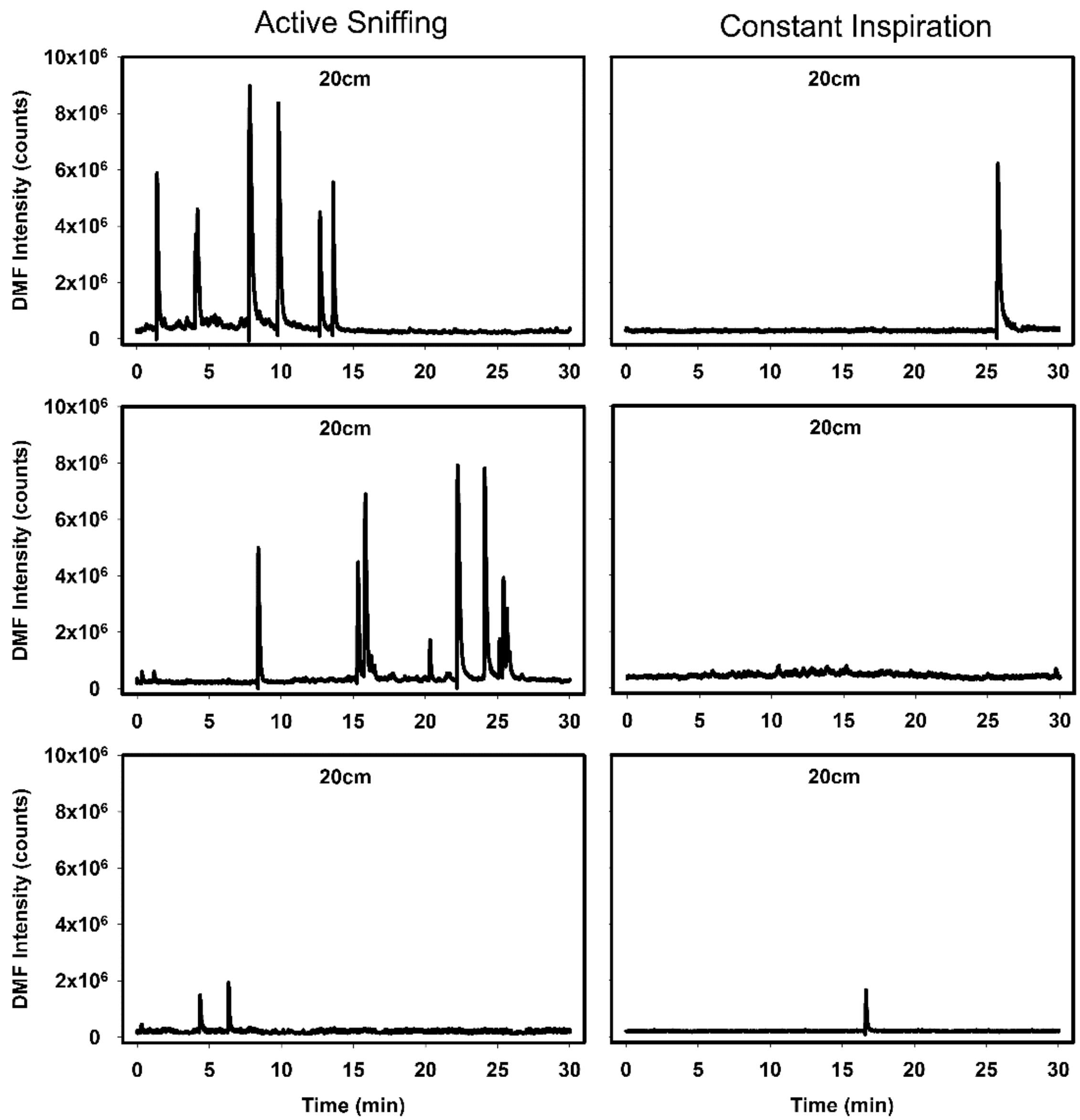

Figure S7. DMF chromatograms for data collected at a distance of 20cm from the vapor source. The case of active sniffing with the dog's nose is the left column and constant inspiration is the right column. On average, more peaks were present in the DMF signal when the artificial nose was sniffing versus only inspiring.

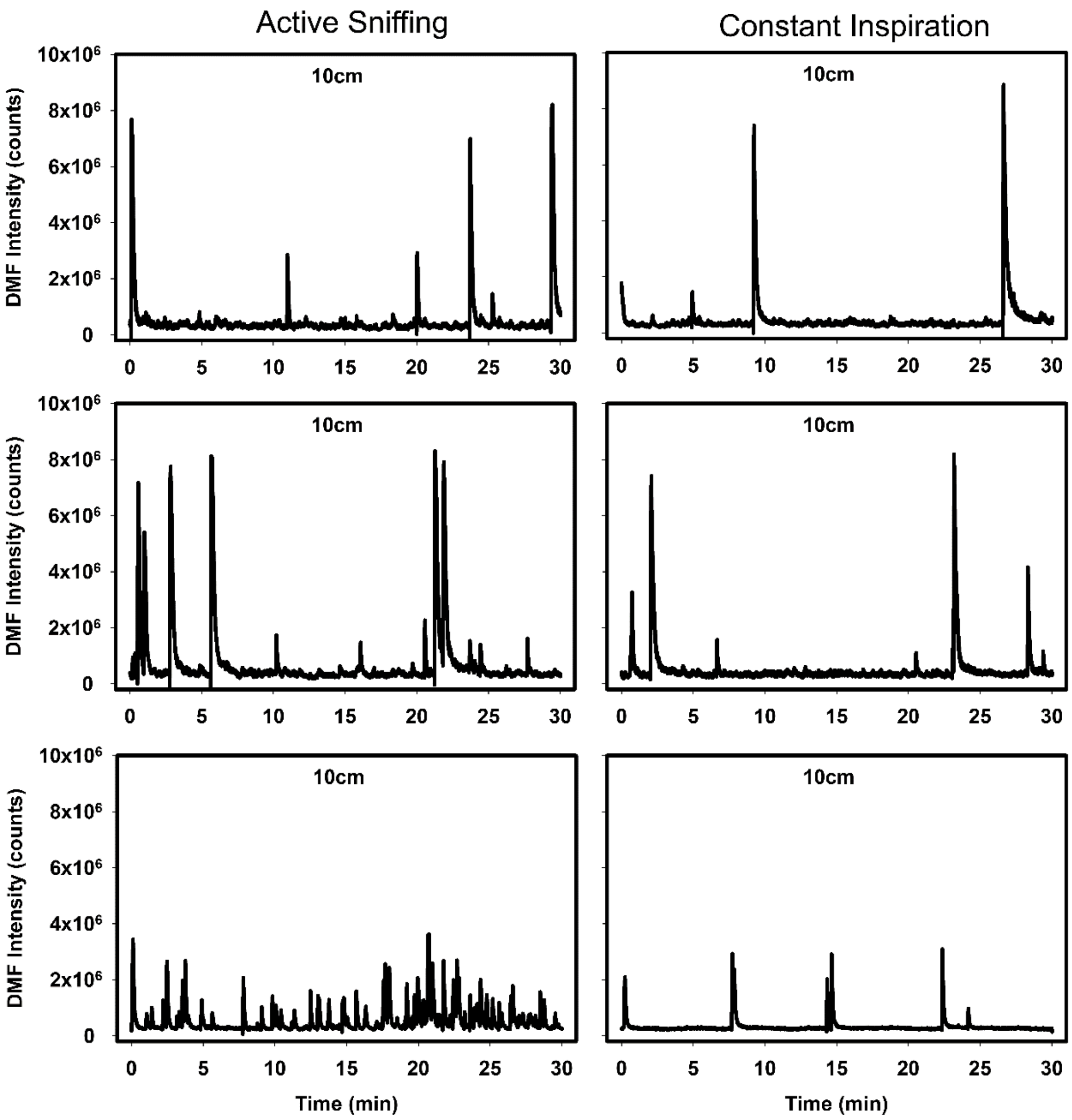

Figure S8. DMF chromatograms for data collected at a distance of 10cm from the vapor source. The case of active sniffing with the dog's nose is the left column and constant inspiration is the right column. On average, more peaks were present in the DMF signal when the artificial nose was sniffing versus only inspiring. The difference between the two cases (sniffing vs. inspiration) is less pronounced at 10cm standoff distance (compared to 20cm).
